# Biliary Outlet Obstruction Due to Pancreatic Calculi in a Post-cholecystectomy Patient

**DOI:** 10.7759/cureus.44328

**Published:** 2023-08-29

**Authors:** Joey Almaguer, Dylan Murray, Matthew Murray, Richard Murray

**Affiliations:** 1 Department of Radiology, Texas Tech University Health Sciences Center School of Medicine, Amarillo, USA; 2 Department of Surgery, University College Dublin, Dublin, IRL; 3 Department of Surgery, Royal College of Surgeons, Dublin, IRL; 4 Department of Diagnostic and Interventional Radiology, Northwest Texas Healthcare System, Amarillo, USA

**Keywords:** cholangiography, cholecystectomy, cholestasis, chronic alcoholism, chronic pancreatitis, pancreatic calculus

## Abstract

Chronic pancreatitis has been shown to cause various pathologies, such as biliary strictures and pancreatic malignancies, which can in turn result in biliary outlet obstruction. However, a pancreatic calculus itself resulting in biliary obstruction has been far less observed. The patient in question had a documented history of chronic alcoholism and received a cholecystectomy decades prior to the onset of cholestatic symptoms. Cholangiography demonstrated no indication of biliary stricture formation, nor was there radiological evidence of pancreatic pseudocyst or malignant formation. CT evidence across a decade of time established a storyline of pancreatic calculi formation, migration, and resultant biliary obstruction. Subsequent endoscopic sphincterectomy, pancreatic calculi removal, and biliary tree stent placement resulted in laboratory value normalization and clinical symptom resolution.

## Introduction

Biliary tree obstruction can have numerous etiologies, including cholelithiasis, biliary strictures, cholangiocarcinoma, biliary cysts, pancreatic adenocarcinoma, biliary atresia, primary biliary, or sclerosing cholangitis, among others [1]. The consequent cholestasis typically results in altered laboratory values, such as total and direct hyperbilirubinemia, transaminitis, and elevated alkaline phosphatase [2]. Amylase and lipase may or may not be elevated in the setting of acute exacerbations of chronic pancreatitis. Clinical manifestations may include jaundice with or without scleral icterus, generalized pruritus, steatorrhea, fat-soluble vitamin deficiency, as well as epigastric/right upper quadrant pain with potential radiation to the back or right shoulder [3]. Diffuse pancreatic calcification is most commonly associated with chronic pancreatitis secondary to chronic alcoholism. Alcohol consumption is thought to increase the viscosity of exocrine pancreatic secretions, which then solidify to form protein plugs within the pancreatic ducts. Calcium carbonate then accumulates distal to the luminal obstruction and precipitates into the pancreatic calcifications observed on radiographic imaging [4]. The following case report describes a patient with chronic alcoholism, chronic calcific pancreatitis, pancreatic calculi formation, and probable migration of pancreatic calculi to the distal common bile duct with resultant biliary outlet obstruction.

## Case presentation

**Figure 1:**
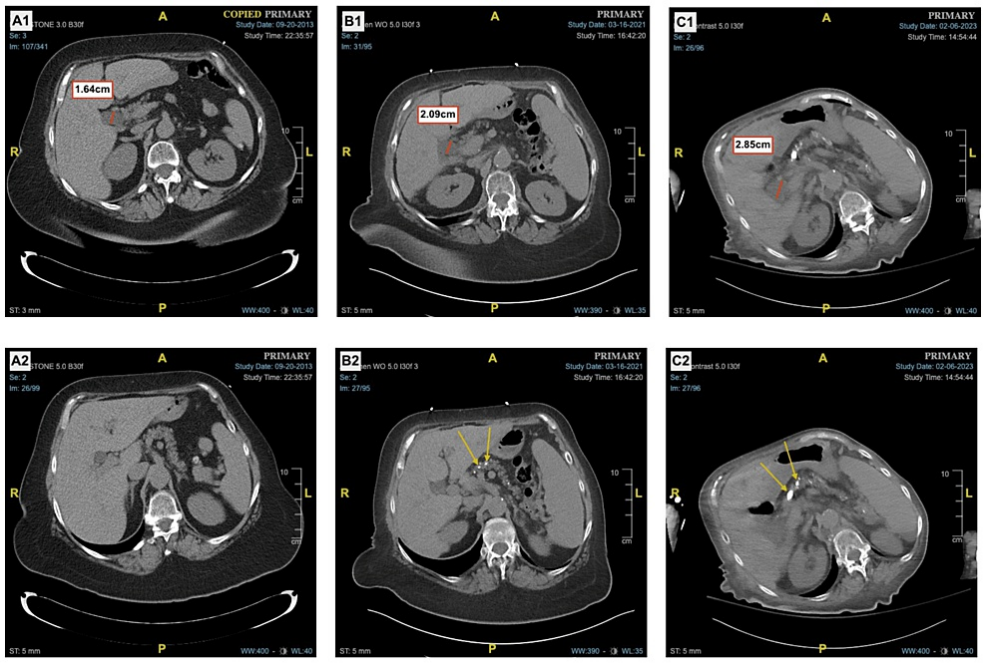
Worsening of post-cholecystectomy biliary dilation and chronic calcific pancreatitis across a span of 10 years (A1/A2) Common bile duct diameter in 2013 was 1.64 cm with little, if any, pancreatic calcifications visualized. (B1/B2) Presumed chronic alcoholism began, resulting in the presence of intraparenchymal calcifications and increased biliary tree dilation. (C1/C2) In February 2023, common bile duct dilation worsened to 2.85 cm with increased intraparenchymal pancreatic calcifications.

A 69-year-old female presented to the emergency department, on March 16, 2021, complaining of unexplained tachycardia, occasional palpitations, dizziness, and concern for a urinary tract infection. Her past surgical history was notable for cholecystectomy more than three decades prior to admission. A CT scan of the abdomen and pelvis revealed liver cirrhosis with mild intrahepatic biliary duct dilation, as well as marked pancreatic parenchymal atrophy with calcification consistent with chronic pancreatitis (Figure 1). There was laboratory evidence of mild transaminitis (aspartate transaminase, or AST, 67 U/L; alanine transaminase, or ALT, 55 U/L) and an elevated alkaline phosphatase (720 U/L) level. After stabilization of her new-onset atrial flutter/fibrillation, she was discharged without any further workup of gastrointestinal abnormalities.

Two years later, on January 10, 2023, the patient was admitted for decompensated liver failure and esophageal variceal bleeding complicated by hemorrhagic shock and anion gap metabolic acidosis. This was the first hospital visit that documented a history of chronic alcoholism with an unspecified starting date. A CT scan of the abdomen and pelvis found common biliary duct dilation with an obstructing stone within the distal common bile duct. Transaminases were increased (AST, 77 U/L; ALT, 76 U/L) with a marked elevation in the alkaline phosphatase (261 U/L) level. Her esophageal varices were stabilized with band ligation and she was subsequently discharged with instructions for an outpatient follow-up with her gastroenterologist regarding her presumed distal choledocholithiasis.

She returned a month later, on February 6, 2023, complaining of chest pain, persistent coffee-ground emesis, and melena, concerning for post-banding ulceration/bleeding. She tested positive for COVID-19 routinely on admission but was on room air and in no respiratory distress. Her labs showed worsening transaminitis (AST, 129 U/L; ALT, 86 U/L), with the alkaline phosphatase level at 900 U/L, total bilirubin at 2.0 mg/dL, hemoglobin of 11.7 g/dL, Hgb A1c of 8.1%, B-type natriuretic peptide (BNP) level of 402.4 pg/mL, and a negative hepatitis panel (Table 1). Amylase and lipase values were not readily ordered due to the perceived precedence of esophageal variceal bleeding and portal hypertension. EKG showed multiple premature ventricular contractions and right bundle branch blocks. Urinalysis was unremarkable and chest X-ray showed pulmonary vascular congestion. Physical examination was significant only for flapping tremor and caput medusae. There was no evidence of steatorrhea or fat-soluble vitamin deficiency. She was treated with pantoprazole, sucralfate, lactulose, rifaximin, propranolol, and ceftriaxone.

**Table 1:** Laboratory value changes across time In 2013, there was no evidence of hepatic pathology reflected in the laboratory values. In 2021, transaminases began to increase along with a precipitous increase in alkaline phosphatase levels, indicating a likely onset of alcohol intake between 2013 and 2021. In January 2023, an increase in the previous values was accompanied by an increase in total bilirubin levels. In February 2023, the highest values in each category were observed. AST, aspartate transaminase; ALT, alanine transaminase

	Total bilirubin (mg/dL)	AST (U/L)	ALT (U/L)	Alkaline phosphatase (U/L)
09/20/2013	0.70	25	21	118
03/16/2021	0.94	67	55	720
01/10/2023	1.18	77	76	261
02/06/2023	2.00	129	86	900

**Figure 2:**
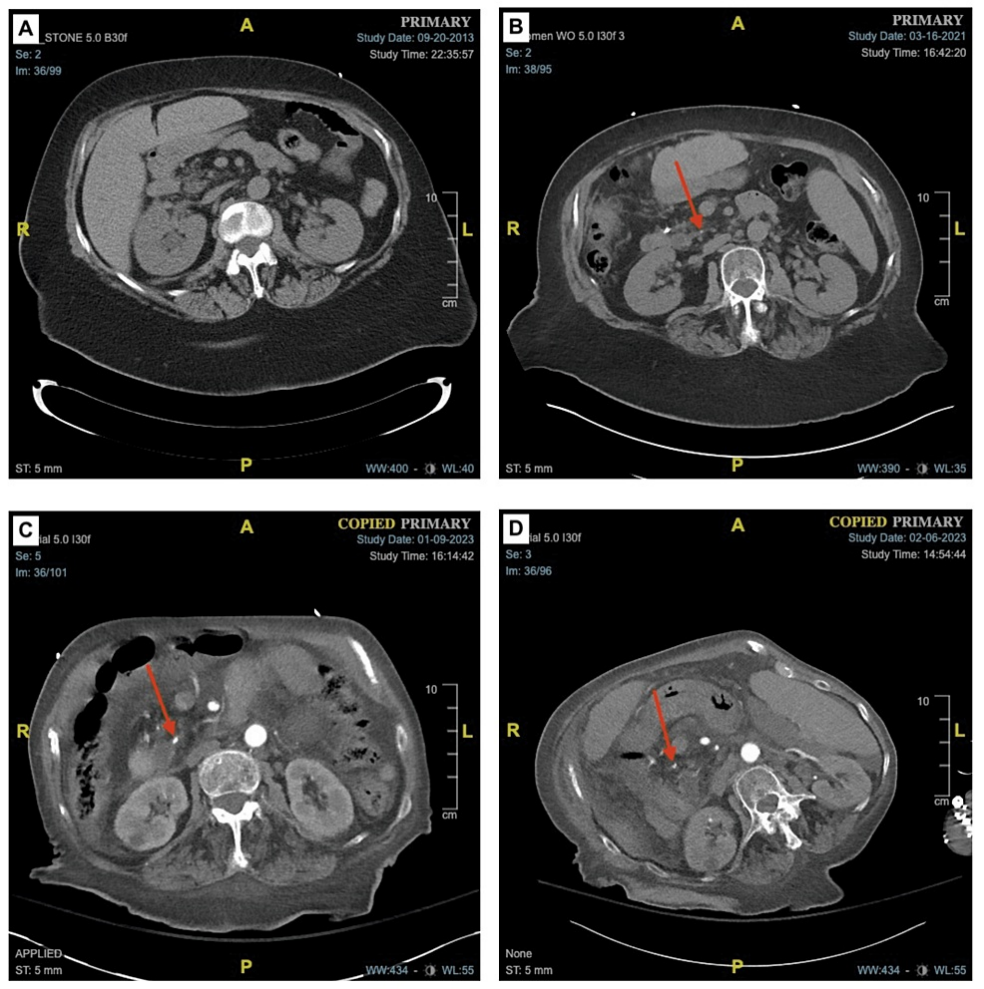
Probable migration of pancreatic calculi to the distal common bile duct, resulting in biliary outlet obstruction (A) The two pancreatic calculi of interest were not observed in 2013. (B) In the 2021 scan, a small calculus can be visualized within the main pancreatic duct. (C) Difficult to differentiate, two abutted calculi migrated more proximally. (D) The two calculi can be seen as visually distinct and at the distal end of the common bile duct.

Esophagogastroduodenoscopy (EGD) showed multiple ulcers in the duodenal bulb and second portion of the duodenum with accompanying duodenitis. There was also portal hypertensive gastropathy and grade 1 esophageal varices with no stigmata of recent bleeding. There was no observed bulging, swelling, or inflammatory changes in the duodenal papilla. Repeat CT of her abdomen and pelvis demonstrated liver cirrhosis with splenomegaly, worsening post-cholecystectomy intrahepatic and extrahepatic biliary duct dilation, and probable interval migration of pancreatic duct calculi (Figure 2). Endoscopic retrograde cholangiopancreatography (ERCP) with cholangiogram revealed a dilated common bile duct with a filling defect; 10-mm sphincterectomy was performed using the Apollo 3AC® Advanced Cannulation Triple Lumen Papillotome (ConMed Corp., New York, USA). The intrabiliary balloon was inflated to 12 mm and swept to remove two stones along with biliary sludge. A Cook Medical Cotton-Leung® 10 French, 5-cm biliary stent (Cook Medical, Bloomington, USA) was placed due to suspicion of an additional common bile duct filling defect. The transjugular intrahepatic portosystemic shunt (TIPS) procedure was postponed due to the increased risk of post-operative TIPSitis given the presence of severe intrahepatic ductal dilation. The patient began to improve with the cessation of hematochezia and melena, as well as the normalization of total bilirubin and transaminases.

Over the past decade, the patient’s CT imaging showed worsening dilation of the intrahepatic and extrahepatic biliary tree, likely due to distal biliary outlet obstruction. The offending pancreatic calculi were observed first in 2021 CT imaging, with proximal migration to the distal end of the common bile duct by March 2023. It is unclear whether the calculi in question began as a single calculus at its inception and subsequently fragmented into two separate calculi or if the calculi were of distinct origin and with time adjoined at the same occlusion point. Gallstone migration into the main pancreatic duct is a documented rare occurrence and is made even more improbable in this case given the patient’s past surgical history of a cholecystectomy decades prior as well as a lack of radiographic evidence of the existing choledocholithiasis in 2013 or 2021. The onset and exacerbation of cholestatic symptoms in the patient correlates with the radiographic evidence in a temporal fashion.

## Discussion

Etiology and demographics

Distal bile duct stenosis occurs in 10%-30% of patients with chronic pancreatitis [5]. However, bile duct obstruction secondary to pancreatic calculi formation is of particular distinction, with only a handful of cases documented in the medical literature [5-12]. Jaundice as a result of a transient increase in bilirubin can be seen, along with a possible progression to cholangitis or acute pancreatitis. The likelihood of chronic pancreatitis manifesting as biliary obstruction likely correlates with the severity and duration of the chronic calcific pancreatitis, as each reported case in one study presented with large calculi and long-standing disease [5]. Pancreatic calculi causing obstruction tend to be larger in size (7-20 mm) in comparison to obstructive biliary stones. Additionally, the larger the stone and longer the common bile duct, the more likely an ampullary obstruction is to take place [7]. As in the presented case, there may be accompanying diabetes mellitus, likely due to pancreatic endocrine insufficiency secondary to long-standing pancreatitis.

Clinical and imaging findings

In imaging studies, contrast CT will likely show the calculus to be at the level of the ampulla with dilation of the biliary tree. A dilated, irregular, and/or tortuous pancreatic duct distal to calculus obstruction may also be evident and correlated with clinical findings of cholestatic disease. Unlike the presented case, duodenoscopy may show an enlarged and elongated papilla, giving endoscopic confirmation of an obstructing calculus at the ampulla. The calculus, which is typically milky-white on gross examination, may sometimes be seen partially exiting through the duodenal ampulla. A composition analysis of pancreatic calculi by infrared spectroscopy will show >95% calcium carbonate composition [7,12].

Differential diagnosis

Distal common bile duct obstruction secondary to pancreatic calculus formation can occur through two processes: occlusion of the shared ampulla of Vater or impingement of the adjacent common biliary duct before it enters the ampulla, resulting in severe stenosis. Other means of physical intraluminal blockage are periductal fibrotic strictures as a result of inflammation-induced pancreatic fibrosis. Alternatively, adjacent mass-effect causing biliary stenosis can result from pseudocyst formation, pancreatic endocrine/exocrine malignancy, or transient pancreatic edema as a result of acute flare-ups in chronic pancreatitis [13].

Treatment and prognosis

In the past, biliary impaction of a pancreatic calculus has been treated with pancreaticoduodenectomy. Other less invasive and more temporary treatment options included percutaneous transhepatic biliary drainage or extracorporeal shock wave lithotripsy (ESWL). Currently, the treatment of choice is endoscopic sphincterectomyor endoscopic pancreatic sphincterectomy by ERCP/needle-knife papillotomy with manual extraction of the offending calculus using grasping forceps, a balloon, basket, or papillotome. Subsequent cholangiogram is performed to reaffirm biliary tree patency and subsequent stent placement may be done to prevent future obstruction. Depending on the size of the impacting calculus and the difficulty with endoscopic removal, ESWL may prove to be beneficial before or after the endoscopic intervention is performed [14]. ESWL may also merit consideration in order to reduce the risk of future re-impaction if there are additional large pancreatic calcifications that cannot be accessed endoscopically [7]. Pancreatic enzyme supplementation and proton pump inhibitors are given to combat resultant pancreatic enzyme and bicarbonate insufficiency.

From the limited cases published, the prognosis appears to be reassuring, with none of the cases describing complications or recurrence of calculus impaction. Most cases report complete pain resolution in all patients who received endoscopic sphincterectomy and biliary decompression [5,10,11]. Long-term benefits from endoscopic intervention can be expected in two-thirds of patients [14,15]. Because chronic calcific pancreatitis is an incurable disease, surgical treatment to remove the biliary obstruction may not resolve symptoms of chronic pancreatitis entirely, nor does it guarantee protection against a future pancreatic calculus re-impaction, especially if the offending behavior, such as chronic alcoholism, persists [16].

## Conclusions

Biliary tree obstruction secondary to migrating pancreatic calculi is a rare occurrence with only a few cases reported in the medical literature. Prior cholecystectomy can help rule out a biliary origin of stone formation in the presence of chronic calcific pancreatitis. In the setting of pancreatic stone formation, interval migration may be seen on radiographic imaging on a long enough timeline basis. If the pancreatic stone occludes the junction of the common bile duct and main pancreatic duct, proximal biliary obstruction can manifest symptomatically. In accordance with previous reports, endoscopic sphincterectomy has proven to be a less invasive, potentially definitive treatment option with promising, long-lasting results.
